# Associations of C-reactive protein isoforms with systemic lupus erythematosus phenotypes and disease activity

**DOI:** 10.1186/s13075-022-02831-9

**Published:** 2022-06-11

**Authors:** Jesper Karlsson, Jonas Wetterö, Maria Weiner, Johan Rönnelid, Rafael Fernandez-Botran, Christopher Sjöwall

**Affiliations:** 1 Department of Biomedical and Clinical Sciences, Division of Inflammation and Infection/Rheumatology, Linköping University, Campus US, 581 85 Linköping, Sweden; 2 Department of Nephrology in Linköping, Department of Health, Medicine and Caring Sciences, Linköping University, Linköping, Sweden; 3 Department of Immunology, Genetics and Pathology, Uppsala University, Uppsala, Sweden; 4 Department of Pathology & Laboratory Medicine, University of Louisville, Louisville, KY USA

**Keywords:** Acute-phase protein, Inflammation, Biomarker, Complement, Pentraxin, Systemic lupus erythematosus, C-reactive protein, Vasculitis

## Abstract

**Background:**

Systemic lupus erythematosus (SLE) is an autoimmune disease characterized by a large production of autoantibodies and deficient clearance of cellular waste. The disease typically oscillates between episodes of elevated disease activity and quiescent disease. C-reactive protein (CRP) is a pentameric acute-phase protein usually reflecting inflammation and tissue damage. However, despite increased inflammation and elevated interleukin-6, the levels of CRP typically remain low or only slightly raised in SLE. Under certain conditions, pentameric CRP (pCRP) can dissociate into its monomeric isoform (mCRP), which mainly has been ascribed pro-inflammatory properties. The present study aims to investigate the potential relationship between pCRP and mCRP, respectively, with disease activity and clinical features of SLE.

**Methods:**

The levels of pCRP and mCRP were measured, by turbidimetry (high-sensitive) and sandwich enzyme-linked immunosorbent assay (ELISA) respectively, in serum samples from 160 patients with SLE and 30 patients with antineutrophil cytoplasmic antibody-associated vasculitis (AAV). Twenty-two of the SLE cases were selected for analysis at two time-points; quiescent disease and active disease. The two CRP isoforms were evaluated in relation to disease activity and clinical features in the two diseases.

**Results:**

Levels of pCRP and mCRP were significantly lower in SLE than AAV (*p* < 0.001) and the ratio of mCRP/pCRP was higher in SLE compared to AAV. The mCRP/pCRP ratio was higher for patients in remission and able to significantly separate between active/quiescent disease in paired, but not in non-paired, samples from patients with SLE. Significant correlations were observed with SLICC/ACR damage index for pCRP levels as well as inversely with the mCRP/pCRP ratio. Lower mCRP levels associated with malar rash.

**Conclusion:**

As the interrelationship between the two isoforms appear to (a) discriminate between quiescent and active SLE and (b) differ between SLE and AAV, our data indicates that the two CRP isoforms could exert contrasting immunological effects and/or reflect different milieus. Given the biological effects of mCRP, it is possible that altered levels may indicate increased opsonization of immune complexes and apoptotic debris, and thereby prevent their deposition outside the reticuloendothelial system and manifestations such as lupus nephritis and lupus-related skin disease.

**Supplementary Information:**

The online version contains supplementary material available at 10.1186/s13075-022-02831-9.

## Introduction

Systemic lupus erythematosus (SLE) is an autoimmune disease with highly heterogenous clinical presentation involving, e.g., skin, joints, and kidneys. Periods of raised disease activity may be followed by prolonged remission, and the disease severity ranges from mild skin and joint manifestations to life-threatening cytopenia, CNS disease, and/or renal involvement [1]. Autoantibody production, deposition of immune complexes (ICs), and complement activation are key components of the SLE pathogenesis. Clearance of dying cells and their constituents is normal and a strictly regulated part of the immune system’s homeostasis. The clearance is eventually carried out mainly by phagocytes, but opsonins, e.g., the acute-phase protein C-reactive protein (CRP), can facilitate this process. In SLE, a number of deficiencies in the waste disposal system has been found, including decreased phagocytic abilities of macrophages and polymorphonuclear cells [2]. Defective clearance is a source of autoantibody generation where the apoptotic cells and cellular debris fails to be eliminated properly, subsequently leading to chromatin and self-DNA being exposed for immune recognition [3]. These autoantibodies combined with soluble antigens form ICs that can accumulate in tissues, recruit complement, and ultimately lead to inflammation and tissue damage [4]. C1q is a complement protein which mitigates activation of the classical complement pathway, partly by CRP binding. Although extremely rare, homozygous deficiency of C1q is a strong risk factor of SLE, which implicates its importance in SLE pathogenesis and supports the waste disposal hypothesis. Furthermore, anti-C1q antibodies are a clinically valuable biomarker associated with SLE disease activity and lupus nephritis [5].

CRP is a highly conserved pattern recognition molecule, usually reflecting inflammation or tissue damage [6]. In contrast to many other inflammatory diseases, CRP appears to be an unreliable marker of raised disease activity in SLE. CRP is liver-derived, and its production is mainly regulated via interleukin-6 (IL-6), but genetic variants, e.g., the polymorphism rs1205, which is overrepresented in SLE, are also of importance for CRP levels [7]. Type I interferons can influence the levels of CRP in the circulation and may therefore be of interest when measuring CRP in serum [8]. However, type I interferons only partly explain the deviating levels of CRP in SLE. Structurally, CRP is a pentahomomeric protein with 5 Identical 206 amino acid subunits. Each subunit has a Ca^2+^-dependent phosphocholine binding site on one side and most likely binds C1q and Fcγ receptors on the other. CRP mediates a variety of pro- and anti-inflammatory biological effects, partly due to its ability to dissociate into a monomeric isoform (mCRP) [9]. CRP recognizes foreign pathogens and initiate clearance by binding to motifs such as phosphocholine, fibronectin, and chromatin, and activates the classical complement system via C1q [8, 10, 11]. The Fcγ receptor recognition aids in binding to Fcγ receptors and thereby activating phagocytotic cells and their clearance of foreign pathogens, apoptotic and damaged cells, and cellular waste [12]. Furthermore, CRP binds both apoptotic and necrotic cells, and low levels of CRP have been proposed to negatively affect the clearance of cellular debris [6, 13]. Since many of the observations do not pinpoint which of the CRP isoforms that are responsible for the certain effect, it is difficult to judge which isoform that associates with specific biological functions. In vitro, the pentameric CRP (pCRP) can dissociate into mCRP when exposed to denaturing conditions as occurs with exposure to membranes and lipoproteins, aqueous surface interfaces (as might occur with stirring and/or freeze/thaw cycles), or when exposed to heat or sufficient concentrations of urea [8, 14, 15]. In some of the above instances, calcium may regulate the dissociation of CRP into the mCRP isoform [16]. mCRP displays neoepitopes which are not exposed in the pentameric native form and asserts pro-inflammatory effector functions which are distinctly different from those of pCRP [17, 18], such as cytokine release, production of reactive oxygen species, and disrupting the alterative complement pathway through binding of lipid rafts and factor H [8, 19, 20]. We and others have shown that autoantibodies to mCRP are commonly found in SLE; especially associating with high disease activity and renal involvement [21, 22]. mCRP also plays an important role in waste disposal mechanisms and inflammatory processes by facilitating opsonization and activation of the classical complement pathway. Furthermore, mCRP has been shown to bind ICs at acidic pH [23], implicating its potential importance in IC-mediated diseases such as SLE. Other data indicate that mCRP in fluid phase (not membrane bound) may have a protective role against tissue damage inflicted by complement by binding of C1q, thereby hindering further activation of the classical complement pathway by C1q [20]. The same finding was obtained with high levels of pCRP (> 150 mg/L) in fluid phase [24]. However, mCRP bound to oxidized LDL has been shown to activate the classical complement pathway [25]. Furthermore, the levels of pCRP have been shown to associate with acquired organ damage in SLE, including the pulmonary and musculoskeletal systems [26]. In the general population, consistently elevated levels of CRP are associated with an increased risk of cardiovascular disease (CVD) such as myocardial infarction and stroke [27], and established CVD therapies has been shown to also lower the CRP concentration [28]. In addition, mCRP has been found in the arterial wall of normal human pulmonary tissue [29]. Although CVD continues to affect patients with SLE at young age, only few studies have been conducted to investigate the association of CVD and CRP specifically in SLE [8]. Recently, Wu et al. showed that the levels of mCRP were higher in patients with antineutrophil cytoplasmic antibody (ANCA)-associated vasculitis (AAV) than in healthy controls, and that these levels were related to CVD in the patients [30].

We hypothesized that inflammation per se may affect the ratios between the isoforms of CRP. Given the important biological function of CRP in relation to elimination of ICs, the present study focused on SLE. Thus, we aimed to evaluate the relationship between pCRP and mCRP and their reflection of disease activity and clinical features in cross-sectional and longitudinal samples from well-characterized patients with SLE. Samples from patients with AAV served as disease controls. To our knowledge, this is the first study to examine the relationship between mCRP and pCRP in SLE.

## Patients and methods

### Study population

The included patients with SLE (*n* = 160) were participants of a prospectively enrolling regional quality register entitled *Clinical Lupus Register in North-Eastern Gothia* (Swedish acronym “KLURING”) based at the University Hospital in Linköping [31]. All patients had a clinical diagnosis of SLE, where 138 (86%) fulfilled the 1982 American College of Rheumatology (ACR-82) classification criteria, and 160 (100%) met the 2012 Systemic Lupus International Collaboration Clinics (SLICC) criteria [32–34]. Disease activity was assessed using the SLE disease activity index 2000 (SLEDAI-2K) [35]. Irreversible organ damage was estimated by the SLICC/ACR damage index (SDI) [33]. Samples from approximately 50% of cases in the KLURING cohort were eligible for the present study to ascertain a broad range of disease activity. The characteristics are detailed in Table 1.

**Table 1:** Characteristics and descriptive data for patients with systemic lupus erythematosus (SLE) and ANCA-associated vasculitis (AAV)

	**SLE (*n* = 160)**	**AAV (*n* = 30)**
**Background variables**		
Age (mean (SD))	59 (18)	66 (11)
Female gender, *n* (%)	139 (87)	14 (47)
**Ongoing pharmacotherapy**		
Glucocorticoids, *n* (%)	110 (69)	22 (73)
Methotrexate, *n* (%)	18 (11)	2 (7)
Mycophenolate mofetil, *n* (%)	22 (14)	1 (3)
Rituximab, *n* (%)	9 (6)	7 (23)
Hydroxychloroquine, *n* (%)	103 (64)	0 (0)
Other immunosuppressants, *n* (%)	21 (13)	0 (0)
**Disease variables**		
Disease duration, years (mean (SD))	21 (11)	0.77 (3.8)
SLEDAI-2K (median (IQR))	4 (1–8)	–
SDI (median (IQR))	1 (0–2)	–
Birmingham Vasculitis Activity Score (median (IQR))	–	14 (12–18)
Microscopic polyangiitis, *n* (%)	–	14 (47)
Granulomatosis with polyangiitis, *n* (%)	–	16 (53)
New-onset (*N*) or established/recurrent (R) disease, *n* (%)	*N*: 5 (3) *R*: 155 (97)	*N*: 27 (90) *R*: 3 (10)
**Clinical laboratory variables**		
Hemoglobin, g/L (mean (SD))	129 (14)	108 (13)
Leukocyte count, 10^9^/L (median (IQR))	6.3 (4.7–8.3)	11.7 (8.2–15.4)
Erythrocyte sedimentation rate, mm/h (median (IQR))	16 (7–33; *n* = 155)	86 (39–100; *n* = 19)
Plasma creatinine, µmol/L (median (IQR))	67 (57–78)	126 (78–287)
Estimated GFR, ml/min/1.73 m^2^ (median (IQR))	79 (62–92)	48 (17–69)
Anti-C1q antibodies, units (median (IQR))	6.4 (3.9–19.9)	–
Anti-C1q antibody positive, *n* (%; ref < 20)	40 (25)	–
MPO-ANCA, IU/mL (median (IQR))	–	0.6 (0.3–55)
MPO-ANCA positive, *n* (%; ref < 3.5)	–	14 (47)
PR3-ANCA, IU/mL (median (IQR))	–	9.8 (0.3–70)
PR3-ANCA positive, *n* (%; ref < 2)	–	16 (53)
^a^Anemia, *n* (%)	36 (23)	26 (87%)
Hematuria, *n* (%)	47 (30; *n* = 155)	23 (82; *n* = 28)
Albuminuria, *n* (%)	29 (18; *n* = 156)	12 (43; *n* = 28)
**Clinical features (ACR–82 defined)**		
1. Malar rash, *n* (%)	59 (37)	–
2. Discoid rash, *n* (%)	29 (18)	–
3. Photosensitivity, *n* (%)	87 (54)	–
4. Oral ulcers, *n* (%)	17 (11)	–
5. Arthritis, *n* (%)	129 (81)	–
6. Serositis, *n* (%)	58 (36)	–
7. Renal disorder, *n* (%)	45 (28)	–
8. Neurologic disorder, *n* (%)	11 (7)	–
9. Hematologic disorder, *n* (%)	97 (61)	–
10. Immunologic disorder, *n* (%)	91 (57)	–
11. IF-ANA, *n* (%)	157 (98)	–

*SLEDAI-2K*, SLE Disease Activity Index 2000; *SDI*, SLICC/ACR damage index; *ESR*, erythrocyte sedimentation rate; *GFR*, glomerular filtration rate; *MPO-ANCA*, myeloperoxidase-antineutrophil cytoplasmic antibodies; *PR3-ANCA*, proteinase 3-antineutrophil cytoplasmic antibodies; *IF-ANA*, antinuclear antibodies analyzed with immunofluorescence; *SD*, standard deviation; *IQR*, interquartile range

^a^Anemia defined as hemoglobin concentration < 117 g/L for women and < 132 g/L for men

Twenty-two of the 160 patients with SLE were selected for paired analysis at two different time-points (visits); one sample with no clinical disease activity (SLEDAI-2K ≤ 4) and one sample with active disease (SLEDAI-2K ≥ 5) were analyzed. The median SLEDAI-2K (with IQR) values for each visit were 1 (0–2) and 12 (9–16), respectively.

AAV patients (*n* = 30), serving as disease controls, were included from the regional vasculitis register based at the University Hospital in Linköping [36]. The patients were recruited between years 2013 and 2020, had a clinical diagnosis of either microscopic polyangiitis (MPA) or granulomatosis with polyangiitis (GPA), and were classified according to the European Medicines Agency (EMA) algorithm [37]. Disease activity was assessed using the Birmingham Vasculitis Activity Score (BVAS) as shown in Table 1 [38].

For the mCRP assessment, 39 healthy controls (HC) with mean age 50 years (range 21–57), 33 females and 6 males, included in previous studies served as comparators [39–42]. For the anti-C1q antibody analyses, 100 anonymized blood donor sera from Uppsala University Hospital served as controls.

### Detection of mCRP and pCRP

Serum concentrations of mCRP were measured using a sandwich enzyme-linked immunosorbent assay (ELISA). Immulon 2HB plates were coated overnight using a goat anti-human mCRP polyclonal antibody diluted in PBS (1:3000) and then blocked for 2 h at room temperature using a solution of 1% bovine serum albumin fraction V (BSA) (Sigma, St. Louis, MO, USA) in PBS-0.01% Tween-20 (blocking buffer). The wells were then washed with PBS-0.01% Tween-20 (washing buffer) and incubated with several concentrations of recombinant human mCRP (0.05–500 ng/mL; as described in Potempa et al. [43]) or serum samples diluted 1:5 in blocking buffer containing 1% normal goat serum for 2 h at room temperature. The wells were then washed three times with washing buffer and incubated with a mouse anti-human mCRP monoclonal antibody (8C10) diluted 1:100 in blocking buffer for 90 min at room temperature. Washing was repeated (3 ×), and the wells were then incubated with a 1:5000 dilution of a goat anti-mouse IgG antibody coupled to horseradish peroxidase (Abcam, Cambridge, UK) in blocking buffer. After a 1-h incubation at room temperature, the plates were washed (3 ×) and a substrate solution (3,3′,5,5′ tetramethylbenzidine [TMB], Sigma) was added to the wells and color allowed to develop. Reactions were stopped by addition of 1 M H_2_SO_4_ and optical density measured at 450 nm. Concentrations of mCRP in the samples were calculated based on the recombinant mCRP standard curve. Controls using purified pCRP at concentrations up to 50 µg/mL did not give any signal above background levels, showing specificity for mCRP.

pCRP in serum was measured using turbidimetry high-sensitive technique (detection limit 0.15 mg/L) at the routine laboratory, Clinical Chemistry laboratory of the University Hospital in Linköping. A cut-off level of 2.0 mg/L was applied according to the clinical routine for cardiovascular risk assessment [44].

### Routine laboratory assessments

Laboratory analyses were carried out at the Clinical Chemistry unit, Linköping University Hospital or the Rudbeck Laboratory, Department of Immunology, Genetics and Pathology, Uppsala University, and included complement components (C3, C4, and C3d by nephelometry; classic hemolytic complement function), erythrocyte sedimentation rate (ESR), hemoglobin concentration, and blood cell counts (leukocytes, lymphocytes, neutrophils, basophils, monocytes, platelets). Urinalysis by urinary dipstick was assessed with regard to urinary erythrocytes (cells/µL) and urinary albumin (g/L). Renal function was measured by estimated glomerular filtration rate (eGFR) based on plasma creatinine [45]. Levels of myeloperoxidase (MPO)- and proteinase 3 (PR3)-ANCA were measured using sensitive fluorescence enzyme immunoassay (FEIA) [46]. Anti-C1q antibodies were analyzed using ELISA (Inova Diagnostics, San Diego, USA) [47].

The levels of C3, C4, and ESR were divided based on reference values from the accredited Clinical Chemistry unit, Linköping. C3 and C4 below 0.7 and 0.13 g/L, respectively, and ESR above 30 mm/h were considered subnormal/abnormal.

### Ethical considerations

All included subjects had provided oral and written informed consent. The study protocol was approved by the Regional Ethics Review Board in Linköping regarding SLE (M75–08/2008) and AAV (2010/205–31).

### Statistical analysis

The data was statistically analyzed using SPSS Statistics 26 (IBM Corp.; Armonk, NY, USA). The data was first tested for normality. Normally distributed data was thereafter examined for outliers. There were no data that fulfilled the requirements for parametrical testing. Testing between non-related groups was carried out using non-parametric Mann–Whitney *U* tests or Kruskal–Wallis test. Non-parametric correlation analyses were performed using Spearman’s rank correlation coefficient test. For comparisons between groups with paired data, Wilcoxon signed rank tests were used. For comparisons between binary data, exact *χ*^2^-test was carried out. Undetectable levels of mCRP were set to half the level of the detection limit (1.25 µg/L). A *p*-value of ≤ 0.05 was considered statistically significant.

## Results

### pCRP and mCRP

**Fig. 1:**
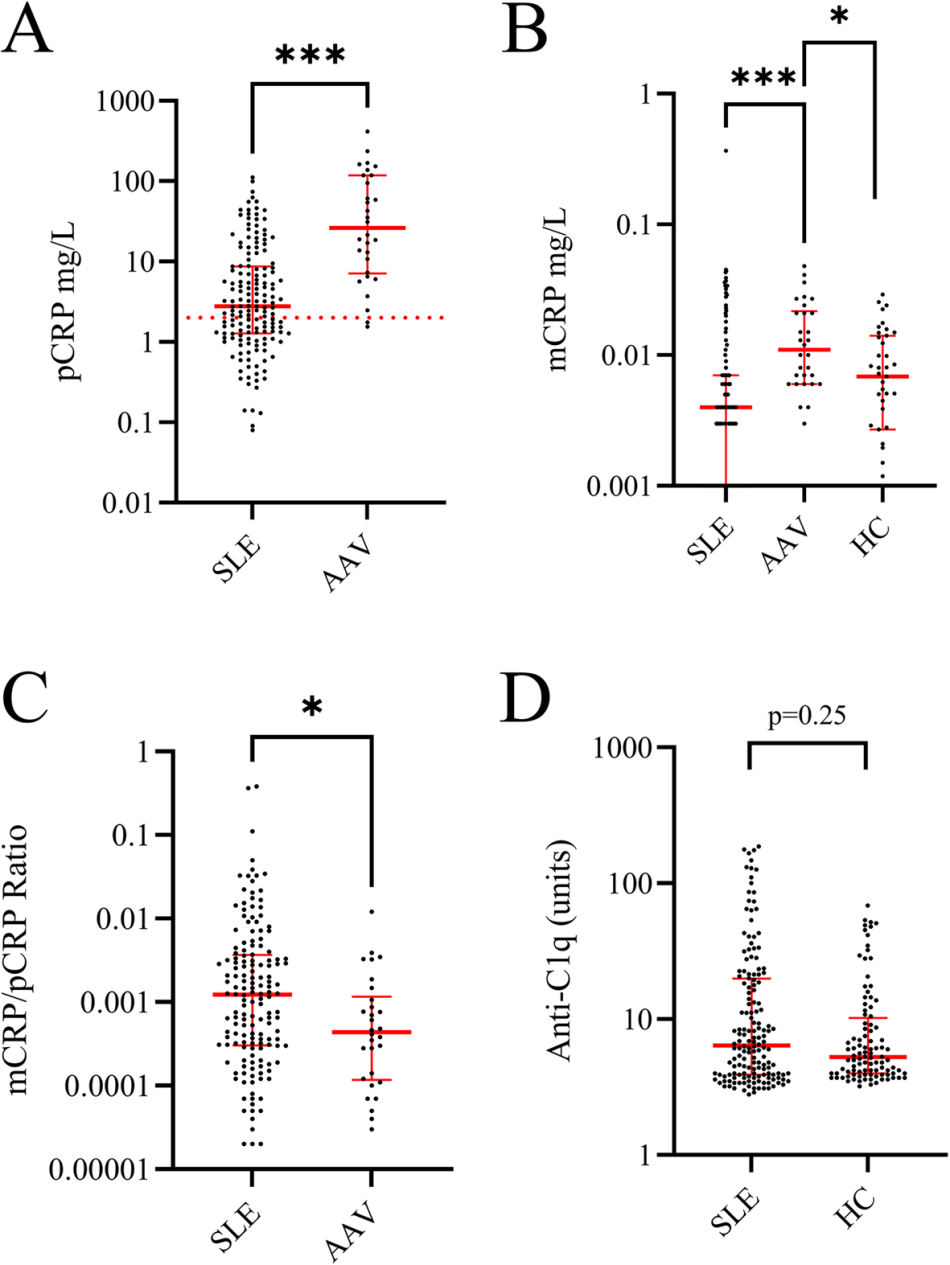
The graphs show serum levels of pentameric C-reactive protein (pCRP; **A**) and monomeric CRP (mCRP; **B**), as well as in mCRP/pCRP ratios (**C**) in systemic lupus erythematosus (SLE; *n* = 160) and ANCA-associated vasculitis (AAV; *n* = 30). Panel **D** illustrates levels of autoantibodies against complement protein 1q (anti-C1q) in SLE as well as in healthy controls (HC; *n* = 100). In addition, panel **B** includes a group of healthy controls (HC; *n* = 39; **B**). The dotted line represents CRP cut-off level applied for cardiovascular risk assessment in clinical routine (2.0 mg/L; **A**) (* = *p* ≤ 0.05, *** = *p* < 0.001)

The median value (with IQR) for the levels of pCRP and mCRP in patients with SLE were 2.8 mg/L (1.3–8.7) and 0.0037 mg/L (0.0013–0.0074) respectively, and 26 mg/L (7.1–118) and 0.011 mg/L (0.0058–0.022) for AAV (Fig. 1). The levels of mCRP and pCRP did not correlate significantly with each other in either SLE (rho =  − 0.002, *p* = 0.98) or AAV (rho = 0.30, *p* = 0.11).

The subjects with SLE had lower levels of both CRP forms than the AAV patients (*p* < 0.001 for both comparisons). In addition, the ratio of mCRP/pCRP showed a significant difference between SLE and AAV (*p* < 0.01; Fig. 1), with mCRP/pCRP ratios showing median values (IQR) 3.7 × 10^−3^ (1.3 × 10^−3^–7.4 × 10^−3^) vs. 4.3 × 10^−4^ (1.2 × 10^−4^–1.2 × 10^−3^).

### CRP in SLE

**Fig. 2:**
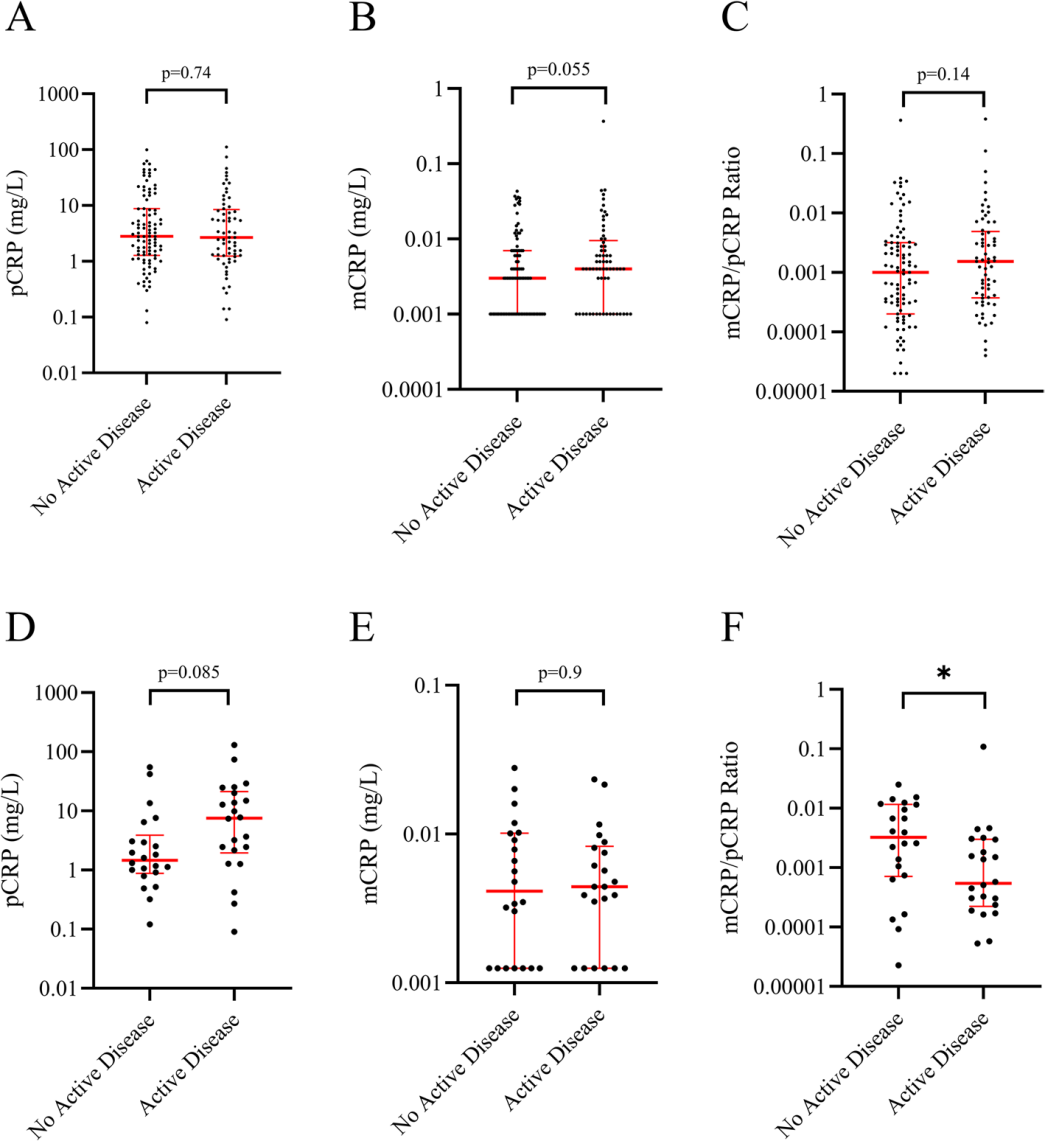
Levels of pCRP (**A**, **D**) and mCRP (**B**, **E**) and ratios of mCRP/pCRP (**C**, **F**) between active and non-active systemic lupus erythematosus. Panels **A**, **B**, and **C** are based on 160 non-paired patient samples whereas **D**, **E**, and **F** represent paired samples from 22 patients (* = *p* ≤ 0.05)

Among the 160 SLE cases, 65 (41%) had active disease (SLEDAI-2K ≥ 5) whereas 95 (59%) were in a quiescent phase of their disease. For paired samples, the ratios of mCRP/pCRP were lower in samples obtained from active compared to non-active disease (*p* ≤ 0.05). However, this comparison did not reach statistical significance in the cross-sectional cohort when samples were divided into active and non-active disease (*p* = 0.14). No significant differences were found regarding the levels of pCRP or mCRP between active disease and non-active disease neither for the paired nor the cross-sectional samples (Fig. 2).

**Fig. 3:**
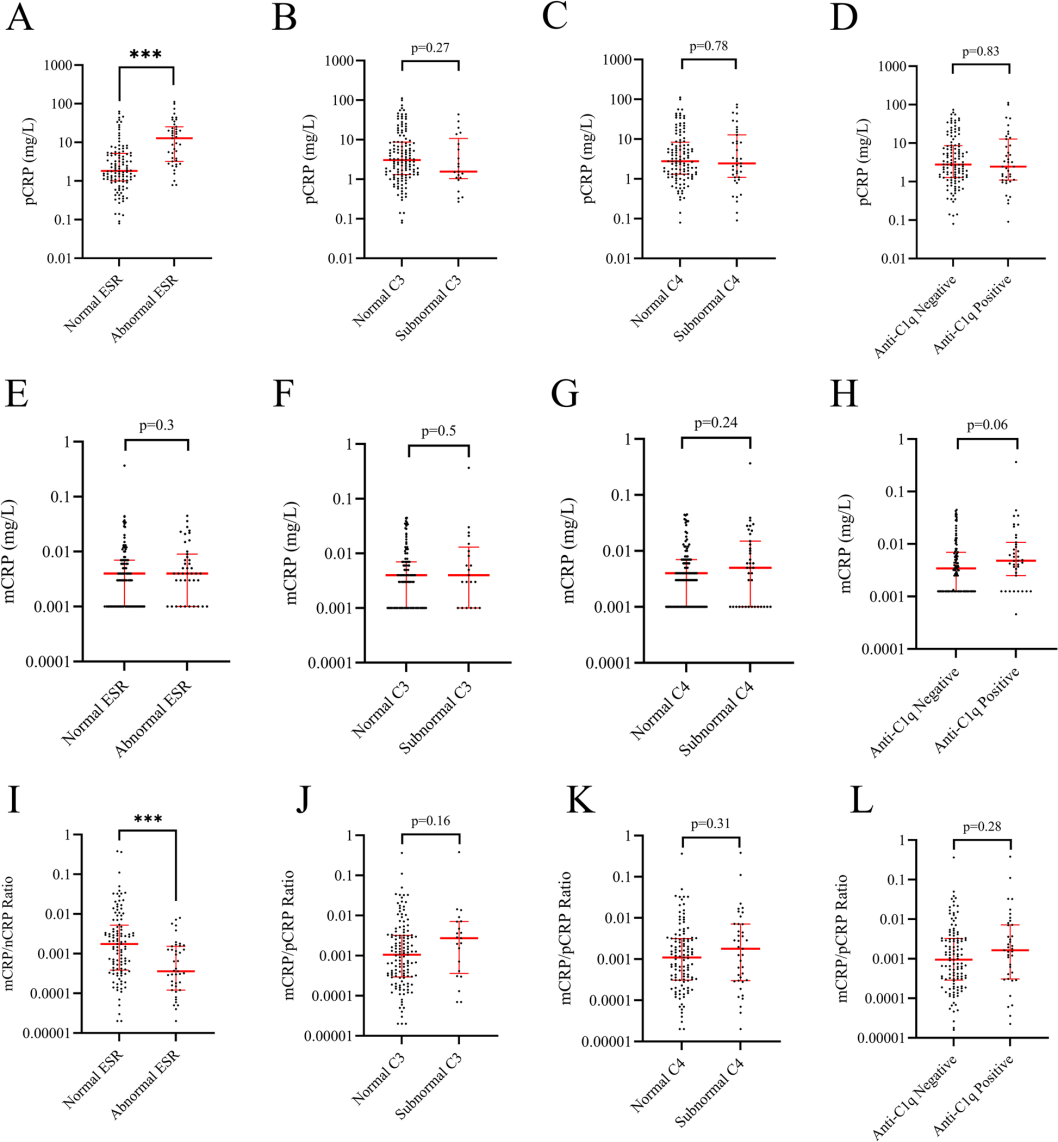
Comparisons of pCRP (**A**–**D**), mCRP (**E**–**H**), and mCRP/pCRP ratios (**I**–**L**) demonstrated between deviating levels of erythrocyte sedimentation rate (ESR), complement protein 3 (C3), C4, and negative/positive anti-C1q autoantibody test in the 160 patients with systemic lupus erythematosus. Abnormal ESR > 30 mm/h; subnormal C3 < 0.7 g/L; subnormal C4 < 0.13 g/L; positive anti-C1q > 20 units (*** = *p* < 0.001)

Patients with normal and abnormal/subnormal levels of ESR, C3, and C4 based on reference intervals were separated into groups, and the levels of pCRP and mCRP as well as the ratios of mCRP/pCRP in each group were compared (Fig. 3). No significant differences were found for patients with normal or subnormal levels of C3 or C4. However, a highly significant difference (*p* < 0.001) was observed for both pCRP and the mCRP/pCRP ratios for patients with normal vs. abnormal ESR.

### CRP in AAV

**Fig. 4:**
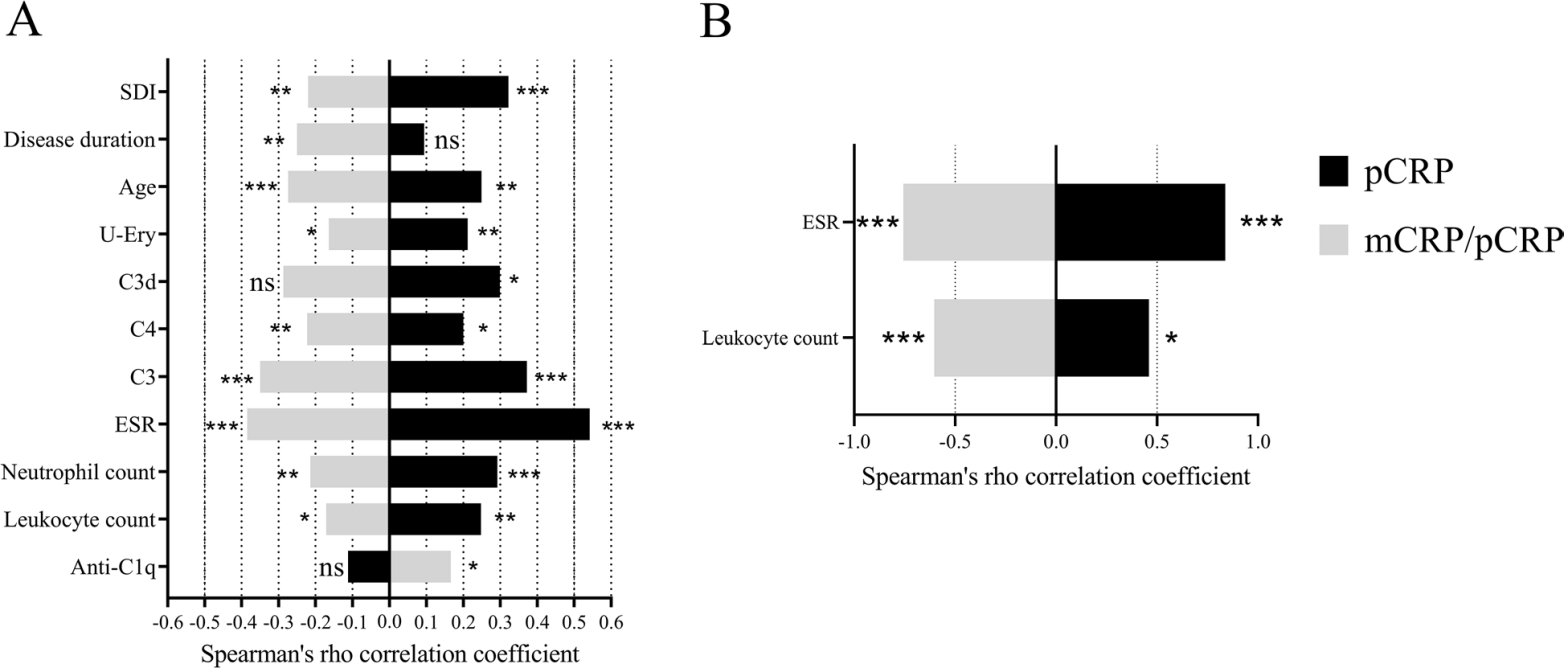
Significant correlations for both pCRP and the mCRP/pCRP ratio based on 160 patients with systemic lupus erythematosus (SLE; **A**) and 30 patients with ANCA-associated vasculitis (AAV; **B**). Black bars represent correlations with pCRP and white bars represent correlations with the mCRP/pCRP ratio. Left of midline represents inverse correlations whereas right of midline represents direct correlations. SDI, SLICC/ACR damage index; U-Ery, Urinary erythrocytes (SLE: *n* = 155); C3d, complement component 3d (*n* = 45); C4, complement protein 4 (*n* = 158); C3, complement protein 3 (*n* = 158); ESR, erythrocyte sedimentation rate (SLE: *n* = 155; AAV: *n* = 19); ns, not significant (* = *p* ≤ 0.05, ** = *p* < 0.01, *** = *p* < 0.001)

Significant differences in the levels of pCRP, mCRP, or the ratio of mCRP/pCRP between samples obtained from patients with MPA and GPA were not observed. Neither did any of the CRP measurements show significant correlations with disease activity assessed by BVAS. The levels of PR3-ANCA were inversely correlated with the mCRP/pCRP ratio (rho =  − 0.42, *p* ≤ 0.05), but did not correlate significantly with pCRP or mCRP. MPO-ANCA levels correlated with the levels of mCRP (rho = 0.39, *p* ≤ 0.05), but not with pCRP or the mCRP/pCRP ratio. Furthermore, the mCRP/pCRP ratio correlated inversely with leukocyte count (rho =  − 0.62, *p* < 0.001) and ESR (rho =  − 0.76, *p* < 0.001, *n* = 19) (Fig. 4). Positive correlations were seen between pCRP levels and leukocyte count (rho = 0.46, *p* ≤ 0.05) as well as for ESR (rho = 0.84, *p* < 0.0001) (Fig. 4), but not with mCRP levels. In addition, mCRP levels correlated inversely with the age of the AAV patients (rho =  − 0.38, *p* ≤ 0.05); this was not observed for pCRP levels, or the mCRP/pCRP ratios.

### Associations between isoforms of CRP and damage accrual in SLE

The levels of pCRP and the mCRP/pCRP ratio correlated significantly with SDI score (rho = 0.33, *p* < 0.001; rho =  − 0.23, *p* =  < 0.01; Fig. 4). Significantly higher levels of pCRP were observed in patients with irreversible damage in the ocular (2.47 mg/L vs. 5.62, *p* ≤ 0.05), neuropsychiatric (2.25 mg/L vs. 6.34, *p* ≤ 0.001), peripheral vascular (2.47 mg/L vs. 7.01, *p* ≤ 0.05), and diabetes (2.67 mg/L vs. 8.41, *p* < 0.01) domains. The mCRP/pCRP ratio was significantly lower in patients with irreversible damage in the neuropsychiatric domain (1.49 × 10^−3^ vs. 4.16 × 10^−4^, *p* < 0.01). No significant change in mCRP levels were found for patients with any type of irreversible damage.

### Associations between isoforms of CRP and clinical features

**Fig. 5:**
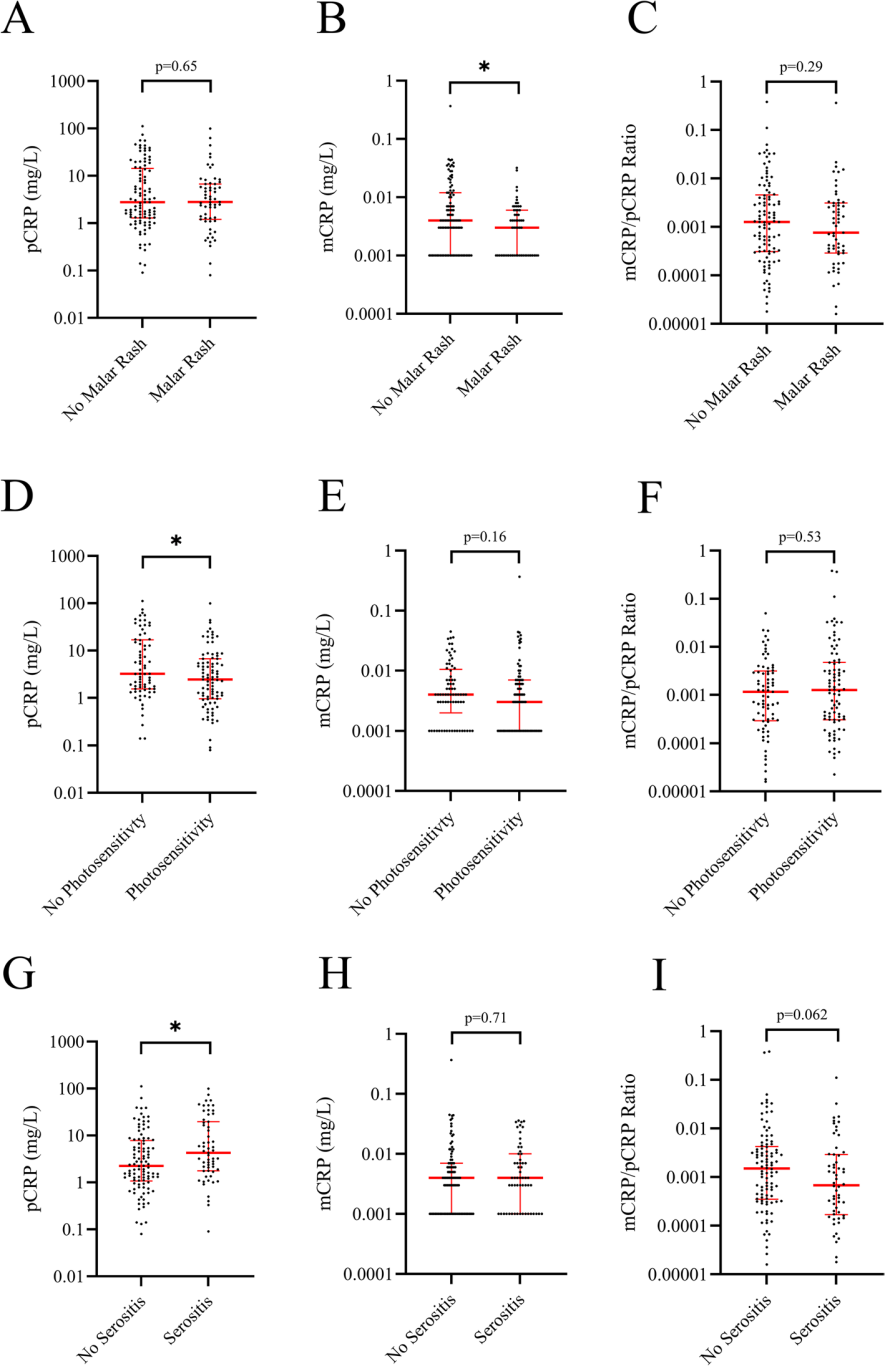
Comparisons of pCRP levels, mCRP levels, and mCRP/pCRP ratio between 160 patients with systemic lupus erythematosus meeting the ACR criteria for malar rash (**A–C**), photosensitivity (**D–F**) and serositis (**G–I**) compared to those that did not (* = *p* ≤ 0.05)

In patients with SLE, the levels of mCRP correlated inversely with disease duration (rho =  − 0.31, *p* < 0.0001) but not with age. When the study population was separated into active/non-active disease, the inverse correlation remained in those with active disease (rho =  − 0.49, *p* < 0.0001, *n* = 65) but not in the non-active. The levels of mCRP were significantly lower among patients meeting the ACR criterion for malar rash (*p* ≤ 0.05). Furthermore, pCRP were decreased in those meeting the criterion for photosensitivity (*p* ≤ 0.05); and higher in patients meeting the criterion for serositis (*p* ≤ 0.05; Fig. 5). Neither the mCRP/pCRP ratio, nor the levels of pCRP and mCRP, were associated with lupus nephritis (Supplementary Fig. 1). In addition, no significant associations between CRP isoforms and any ongoing medication were observed except for the prescribed corticosteroid dose, which significantly correlated with pCRP in SLE (rho = 0.26, *p* = 0.01), but not in AAV.

### Associations between CRP isoforms and laboratory features

In SLE, levels of mCRP in the patient group in remission correlated inversely with classical complement function (rho =  − 0.47, *p* ≤ 0.05, *n* = 22) and positively with basophil count (rho = 0.28, *p* < 0.01, *n* = 94). The pCRP levels were significantly higher in patients with anemia compared to those with hemoglobin concentrations within the reference limits (2.56 mg/L vs. 3.77, *p* ≤ 0.05), but no significant difference was detected for mCRP levels or the mCRP/pCRP ratio.

In AAV, there was no significant difference in pCRP levels or the mCRP/pCRP ratio between patients with anemia and those with hemoglobin concentration within the reference limit; however, significantly lower levels of mCRP were found in patients with anemia (0.024 mg/L vs. 0.0088, *p* ≤ 0.05). Differences in pCRP, mCRP, and mCRP/pCRP ratio were analyzed with regard to presence of proteinuria and hematuria, and with impaired renal function (eGFR < 60 mL/min/1.73 m^2^).

Significantly higher pCRP levels were found in SLE with hematuria compared to those without (*p* ≤ 0.05; Supplementary Fig. 1). The levels of mCRP were higher in AAV patients with albuminuria compared to those without (Supplementary Fig. 2). In addition, the levels of anti-C1q autoantibodies correlated weakly with the ratio of mCRP/pCRP (Fig. 4). No significant difference was found for anti-C1q autoantibody levels between SLE and healthy controls (Fig. 1D).

## Discussion

Typically, pCRP levels remain low or only slightly elevated in SLE flares even if IL-6 is elevated, and these levels are often higher during infections than lupus flares [7, 8, 48]. Thus, pCRP is considered an unreliable marker of increased inflammation in SLE. To our knowledge, the present study is the first to evaluate the two isoforms of CRP in SLE.

In line with previous findings, we show that pCRP did not discriminate between active and non-active SLE. However, the mCRP/pCRP ratio met statistical significance in paired samples. Previously, it has been shown that mCRP possesses a number of distinct biological effects and can be of importance in relation to IC elimination via the reticuloendothelial system [23, 49]. The latter is of high relevance in SLE pathogenesis where extra-hepatic deposition of ICs is prevalent. Furthermore, it has been demonstrated that CRP together with C1q and dsDNA co-localize with IgG in electron-dense deposits of proliferative lupus nephritis [50], implicating that antibodies targeting dsDNA, CRP, and C1q are involved in the SLE pathogenesis. Presence of anti-CRP antibodies in SLE has been reported in varying frequencies by several groups [8]. These antibodies fluctuate over time but have consistently been found to associate with lupus nephritis and have the ability to bind apoptotic material and induce pro-inflammatory responses [21, 51, 52]. In addition, no significant correlation seem to exist between anti-CRP antibodies and CRP levels in serum [53]. Anti-mCRP antibodies have been shown to correlate with SLE disease activity [54]. Anti-CRP antibodies show a resemblance with anti-C1q antibodies, which bind to conformationally altered C1q and are strongly associated with lupus nephritis [5, 55]. The deposition of anti-C1q in glomeruli has previously been suggested to reveal neoepitopes not exposed elsewhere [5]. Prior observations of the antigen specificity have revealed that the anti-CRP antibodies found in SLE are directed towards mCRP, not pCRP, and that isolated ICs do not give rise to false positive anti-CRP [54, 56].

Given the recent publication by Wu et al. [30] on mCRP and pCRP in AAV, we decided to include a group of well-characterized patients with AAV as disease controls. Not surprisingly, the pCRP and mCRP levels in SLE were lower than in AAV. The ratio of mCRP/pCRP also differed, showing higher ratios in SLE compared to AAV. In contrast to Wu et al. [30], mCRP in our study did not correlate with BVAS in AAV patients (rho = 0.17, *p* = 0.37). However, in general, the patients with AAV included herein overall had less severe disease, which could indicate that the levels of mCRP is greater at even higher degree of disease activity than the patients in this study had. Comparisons between this study and the study by Wu et al. [30] shows that the level of mCRP in AAV differs greatly, where the patients herein had considerably lower levels. However, the mCRP levels in our study are more approximate to those reported by Chen et al. [57], and in line with other autoimmune disorders and with patients of high CRP [58–60]. The levels of mCRP were shown to correlate with age in AAV, while also inversely correlate with disease duration in SLE. Interestingly, this correlation was stronger in the SLE patients with an active disease profile, while the correlation in the patients without active disease was non-significant. The in-house ELISA used for detecting mCRP in serum displayed highly specific results using the 8C10 clone and has not been previously published (Supplementary Fig. 3).

Data on mCRP have been reported in AAV [30, 57], autoimmune dermatological disorders [60], bacterial infections [57, 59], CVD [58], healthy volunteers [30, 58, 60], and in a limited number of patients with SLE and rheumatoid arthritis [57]. Increased pCRP has been reported to associate with organ damage [26]. Similarly, in the present study we observed significant correlations between SDI and pCRP as well as with mCRP/pCRP ratio. Furthermore, SLE patients without malar rash had higher levels of mCRP than those meeting that ACR criterion. pCRP levels were higher in those with serositis, and both pCRP and mCRP were associated with skin manifestations (photosensitivity and malar rash). Patients with other autoimmune skin disorders have been reported to show elevated levels of mCRP compared to healthy controls [60]. Previously, mCRP has been shown to activate neutrophils, monocytes, and platelets [8, 61]. In the present study however, the levels of mCRP were not correlated with either neutrophil, monocyte, or platelet counts. Although the biological function of mCRP entails activation of the classical complement pathway [62], disruption of the alternative complement pathway [17], facilitation of opsonization [63], and activation of endothelial cells [17], in our hands, the levels of mCRP did not correlate significantly with the levels of complement proteins (C3, C4, or C3d).

When planning this study, we initially hypothesized that the mCRP/pCRP ratio could reflect disease activity or clinical features in SLE. Some associations were indeed observed, but the mCRP/pCRP ratio seems to predominantly be influenced by the levels of pCRP.

## Conclusions

As the interrelationship between the two isoforms appear to (a) discriminate between quiescent and active SLE, and (b) differ between SLE and AAV, our data indicates that the two CRP isoforms could exert contrasting immunological effects and/or reflect different milieus. Given the biological effects of mCRP, it is possible that altered levels may indicate increased opsonization of ICs and apoptotic debris, and thereby preventing their deposition outside the reticuloendothelial system and manifestations such as lupus nephritis and lupus-related skin disease.

## Supplementary Information

**Additional file 1:** **Supplementary Figure 1.** Comparisons of pentameric C-reactive protein (pCRP) (A–D), monomeric (m)CRP (E–H), and mCRP/pCRP ratio (I–L) in 160 patients with systemic lupus erythematosus with lupus nephritis vs. no nephritis (A, E, I), normal vs. subnormal estimated glomerular filtration rate (eGFR) (B, F, J), presence vs. absence of hematuria (C, G, K) and presence vs. absence of albuminuria (D, H, L). Abnormal eGFR <60 mL/min/1.73m^2^. (* = *p*≤0.05).

**Additional file 2:** **Supplementary Figure 2.** Comparisons of pentameric C-reactive protein (pCRP) (A–C), monomeric (m)CRP (D–F), and mCRP/pCRP ratio (G–I) demonstrated between normal vs. subnormal estimated glomerular filtration rate (eGFR) (A, D, G), presence vs. absence of hematuria (B, E, H) and presence vs. absence albuminuria (C, F, I) in patients with ANCA-associated vasculitis. Abnormal eGFR <60 mL/min/1.73m^2^. (* = *p*≤0.05).

**Additional file 3:** **Supplementary Figure 3.** Standard curves and reactivity of the monoclonal detection antibody 8C10 towards monomeric C-reactive protein (mCRP) and pentameric (p)CRP: (A) standard curve for 8C10 antibody with mCRP; (B) reactivity of 8C10 antibody with pCRP; and (C) standard curve for samples in the present study.

## Data Availability

The datasets used and/or analyzed during the current study are available from the corresponding author on reasonable request.

## Abbreviations

SLE: Systemic lupus erythematosus
CRP: C-reactive protein
mCRP: Monomeric C-reactive protein
pCRP: Pentameric C-reactive protein
AAV: Antineutrophil cytoplasmic antibody-associated vasculitis
ICs: Immune complexes
IL-6: Interleukin-6
CVD: Cardiovascular disease
ANCA: Antineutrophil cytoplasmic antibody
KLURING: Swedish acronym for Clinical Lupus Register in North-Eastern Gothia
ACR-82: 1982 American College of Rheumatology classification criteria
SLICC: 2012 Systemic Lupus International Collaboration Clinics criteria
SLEDAI-2K: SLE disease activity index 2000
SDI: SLICC/ACR damage index
IQR: Interquartile range
MPA: Microscopic polyangiitis
GPA: Granulomatosis with polyangiitis
EMA: European Medicines Agency
BVAS: Birmingham Vasculitis Activity Score
HC: Healthy controls
ELISA: Enzyme-linked immunosorbent assay
BSA: Bovine serum albumin
TMB: Tetramethylbenzidine
C3: Complement component 3
C4: Complement component 4
C3d: Complement component 3d
C1q: Complement component 1q
ESR: Erythrocyte sedimentation rate
eGFR: Estimated glomerular filtration rate
MPO: Myeloperoxidase
PR3: Proteinase 3
FEIA: Fluorescence enzyme immunoassay
IgG: Immunoglobulin G
