# Supplementary figures and images for: Associations of C-reactive protein isoforms with systemic lupus erythematosus phenotypes and disease activity
Source: Arthritis Res Ther. 2022 Jun 11;24:139. doi: 10.1186/s13075-022-02831-9 (PMC9188243; doi:10.1186/s13075-022-02831-9)

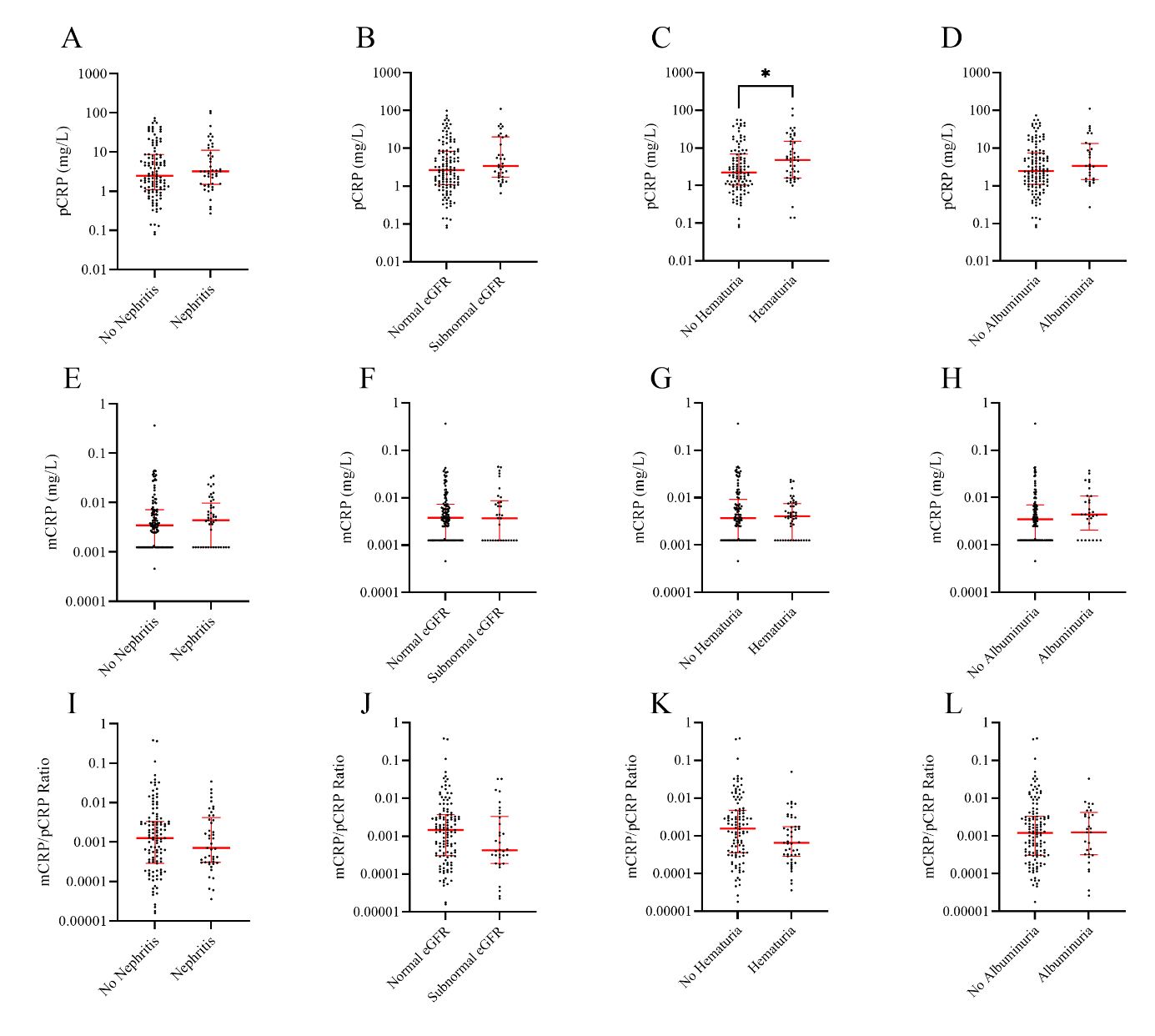

Supplement: Supplementary file 1 — Additional file 1: Supplementary Figure 1. Comparisons of pentameric C-reactive protein (pCRP) (A–D), monomeric (m)CRP (E–H), and mCRP/pCRP ratio (I–L) in 160 patients with systemic lupus erythematosus with lupus nephritis vs. no nephritis (A, E, I), normal vs. subnormal estimated glomerular filtration rate (eGFR) (B, F, J), presence vs. absence of hematuria (C, G, K) and presence vs. absence of albuminuria (D, H, L). Abnormal eGFR <60 mL/min/1.73m2. (* = p≤0.05). [file 13075_2022_2831_MOESM1_ESM.docx]

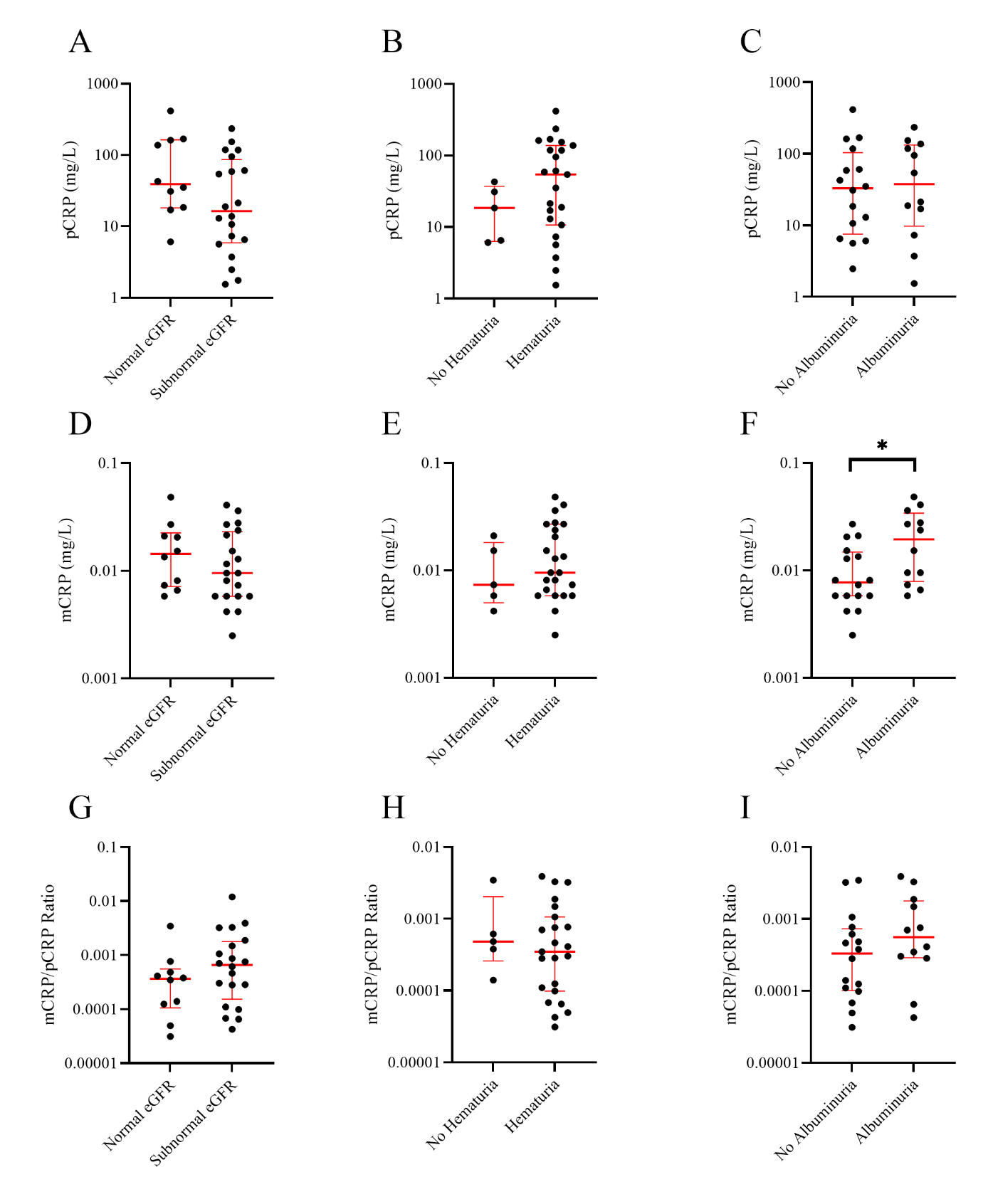

Supplement: Supplementary file 2 — Additional file 2: Supplementary Figure 2. Comparisons of pentameric C-reactive protein (pCRP) (A–C), monomeric (m)CRP (D–F), and mCRP/pCRP ratio (G–I) demonstrated between normal vs. subnormal estimated glomerular filtration rate (eGFR) (A, D, G), presence vs. absence of hematuria (B, E, H) and presence vs. absence albuminuria (C, F, I) in patients with ANCA-associated vasculitis. Abnormal eGFR <60 mL/min/1.73m2. (* = p≤0.05). [file 13075_2022_2831_MOESM2_ESM.docx]

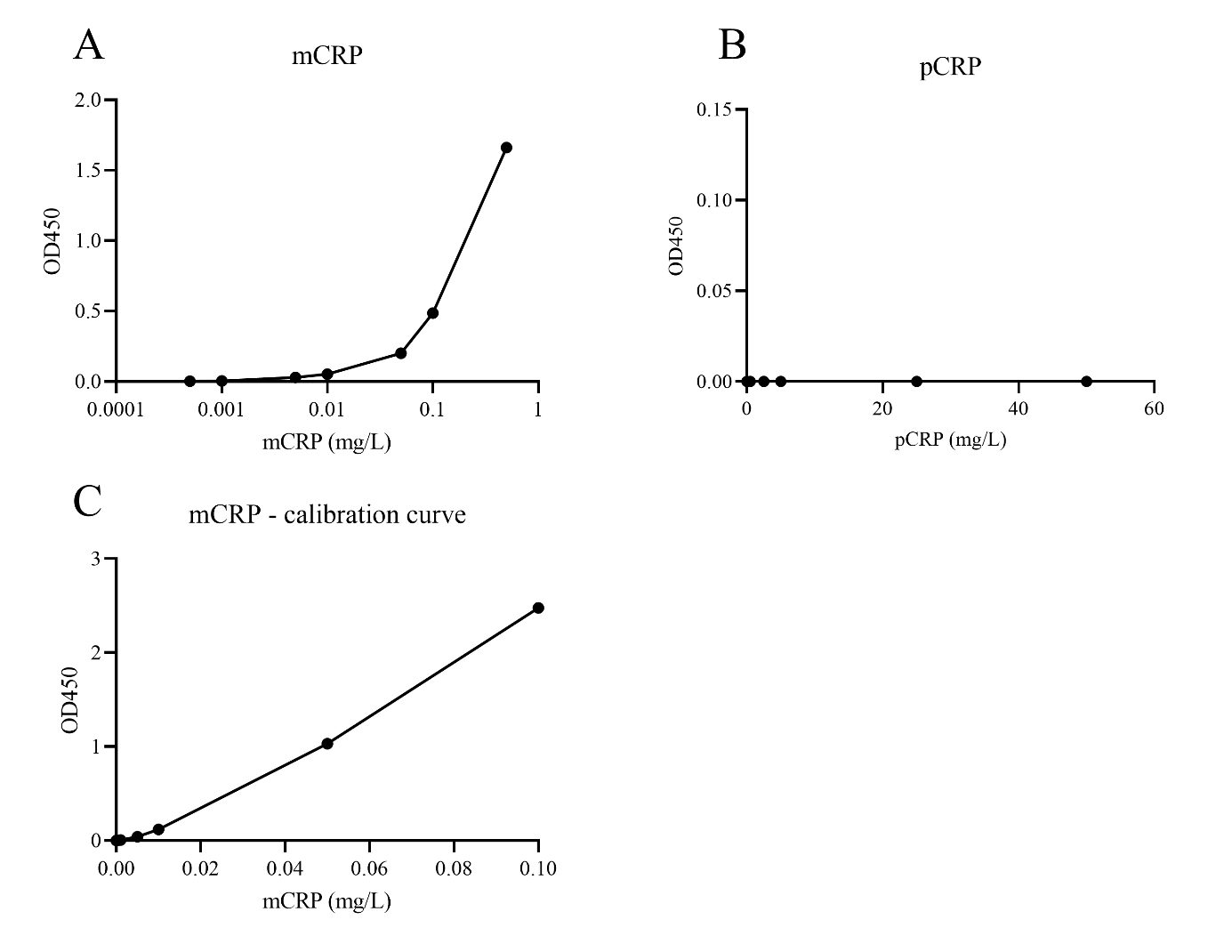

Supplement: Supplementary file 3 — Additional file 3: Supplementary Figure 3. Standard curves and reactivity of the monoclonal detection antibody 8C10 towards monomeric C-reactive protein (mCRP) and pentameric (p)CRP: (A) standard curve for 8C10 antibody with mCRP; (B) reactivity of 8C10 antibody with pCRP; and (C) standard curve for samples in the present study. [file 13075_2022_2831_MOESM3_ESM.docx]
